# Mitophagy—A New Target of Bone Disease

**DOI:** 10.3390/biom12101420

**Published:** 2022-10-04

**Authors:** Zhipeng Zeng, Xuchang Zhou, Yan Wang, Hong Cao, Jianmin Guo, Ping Wang, Yajing Yang, Yan Wang

**Affiliations:** 1 School of Sport Medicine and Rehabilitation, Beijing Sport University, Beijing 100084, China; 2 School of Kinesiology, Shanghai University of Sport, Shanghai 200438, China; 3 Cancer Hospital & Shenzhen Hospital, Chinese Academy of Medical Sciences and Peking Union Medical College, Shenzhen 518116, China; 4 Department of Rehabilitation, First Affiliated Hospital of Gannan Medical University, Ganzhou 341000, China; 5 School of Physical Education and Sports Science, Lingnan Normal University, Zhanjiang 524048, China

**Keywords:** mitophagy, osteoarthritis, intervertebral disc degeneration, osteoporosis, osteosarcoma

## Abstract

Bone diseases are usually caused by abnormal metabolism and death of cells in bones, including osteoblasts, osteoclasts, osteocytes, chondrocytes, and bone marrow mesenchymal stem cells. Mitochondrial dysfunction, as an important cause of abnormal cell metabolism, is widely involved in the occurrence and progression of multiple bone diseases, including osteoarthritis, intervertebral disc degeneration, osteoporosis, and osteosarcoma. As selective mitochondrial autophagy for damaged or dysfunctional mitochondria, mitophagy is closely related to mitochondrial quality control and homeostasis. Accumulating evidence suggests that mitophagy plays an important regulatory role in bone disease, indicating that regulating the level of mitophagy may be a new strategy for bone-related diseases. Therefore, by reviewing the relevant literature in recent years, this paper reviews the potential mechanism of mitophagy in bone-related diseases, including osteoarthritis, intervertebral disc degeneration, osteoporosis, and osteosarcoma, to provide a theoretical basis for the related research of mitophagy in bone diseases.

## 1. Introduction

Bone homeostasis depends on coupled bone formation and bone resorption, which ensures normal bone development and basic physiological functions. On the one hand, cartilage containing chondrocytes gradually ossifies to form normal bone tissue; on the other hand, osteoblasts and osteoclasts restrict each other to maintain the stability of bone metabolism [1]. Disturbance or abnormality in the dynamic balance between bone resorption and bone formation can lead to the occurrence of various diseases, including osteoarthritis (OA), intervertebral disc degeneration (IVDD), osteoporosis (OP), and osteosarcoma (OS). Therefore, understanding the cellular life activities and potential regulatory mechanisms in the process of bone metabolism is of great significance for the prevention and treatment of bone-related diseases.

Autophagy, a membrane-dependent mechanism for the turnover of subcellular components, is a highly conserved catabolic process in eukaryotic cells [2]. The main function of autophagy is to degrade aged or damaged organelles and remove unnecessary macromolecules or pathogens such as misfolded proteins, damaged mitochondria, and peroxisomes while providing cells with energy and nutrients through the lysosomal mechanism [3]. Autophagy is highly regulated to maintain a balance between the synthesis, degradation, and subsequent recycling of cellular products that are critical for cell growth, survival, differentiation, and homeostasis [4]. Appropriate levels of autophagy can protect cells from pathological or physiological damage, while excessive or reduced autophagy can trigger apoptosis [5]. Cumulative studies have shown that dysregulation of autophagy is involved in the pathogenesis of a variety of human diseases, including cancer, metabolic disease, neurodegenerative disease, and cardiovascular disease [6]. Autophagy includes macro-autophagy, mini-autophagy, and chaperone-mediated autophagy. Autophagy is considered nonspecific. The selective degradation of mitochondria by the autophagic mechanism is called mitophagy, which is a type of macro-autophagy [7]. As the main site of adenosine triphosphate (ATP) supply, mitochondria are rapidly transformed into death-promoting organelles under abnormal conditions. Mitophagy is one of the major mitochondrial quality control processes that maintain optimal mitochondrial integrity. Mitophagy, as a specialized form of autophagy, maintains mitochondrial homeostasis by eliminating damaged organelles and excess proteins and reducing cellular stress caused by noxious stimuli [8]. Mitophagy is a normal physiological activity that occurs under healthy conditions to maintain cellular homeostasis. However, under pathological or specific physiological conditions, the level of mitophagy can also be altered to promote various diseases, including heart disease [9], neurodegenerative diseases [10], and kidney disease [11]. Therefore, precise regulation of mitophagy may be a more specific and promising strategy for diseases associated with abnormal mitophagy. In recent years, increasing evidence has shown that abnormal levels of mitophagy can disrupt the balance of bone metabolism and play a key role in bone-related diseases, suggesting that mitophagy-based intervention therapy may be a promising therapeutic strategy for bone diseases [12]. However, the current study of mitophagy in bone disease is still in its early stages. An in-depth understanding of the specific mechanisms by which mitophagy regulates bone-related diseases can help develop more effective therapeutic strategies. In this paper, by reviewing the relevant research literature on mitophagy and bone-related diseases, we summarize the potential mechanism of mitophagy regulating bone diseases, which provides a reference for further research on mitophagy in bone-related diseases.

## 2. An Overview of Mitophagy

Mitochondria play a key role in the production of ATP, which provides more than 90% of the energy for cells [13]. Mitochondrial DNA integrity and repair capacity are impaired in response to stress, such as the increase in mitochondrial transmembrane potential (MMP), oxidative stress, and iron starvation. If the damage exceeds the membrane potential across the inner mitochondrial membrane, dysfunctional mitochondria are cleared through the autophagic pathway, which is known as mitophagy [14]. Mitophagy is a self-protective mechanism of cells that removes damaged or dysfunctional mitochondria through the targeted phagocytosis and destruction of mitochondria by the autophagic apparatus, which is considered to be a major mechanism of mitochondrial quality control to maintain the stability of mitochondrial quantity and function in cells [15]. Mitophagy is associated with fission events that maintain a healthy mitochondrial pool. Therefore, basal levels of mitophagy are critical, as it maintains cellular homeostasis and matches mitochondrial numbers to metabolic demands, thereby protecting cells against dysfunctional mitochondria [16]. Under stress conditions, mitophagy is activated, resulting in enhanced mitophagy as an early response to promote cell survival by removing damaged mitochondria [17]. However, an excessive increase in caspases inhibits the induction of mitophagy, leading to the accumulation of damaged mitochondria and, ultimately, cell death [18]. Therefore, it can be considered that, on the one hand, mitophagy can protect cells from apoptotic damaged mitochondria due to mitophagy removal leading to mitochondrial apoptosis; on the other hand, excessive mitophagy can also induce apoptosis due to lack of mitochondria leading to insufficient energy supply in cells [19], suggesting that moderate mitophagy levels are important for cell survival.

In recent years, increasing studies have shown that moderate mitophagy induction has favorable effects on the treatment of various diseases, including atherosclerosis [20], acute kidney injury [21], and traumatic brain injury [22], suggesting that human diseases are closely related to mitophagy [23]. Several mechanisms of mitophagy have been identified. In general, mitophagy includes Parkin RBR E3 ubiquitin protein ligase (PRKN)-dependent and PRKN-independent pathways [24], of which PRKN-dependent mitophagy has been the most widely studied [25]. PRKN-dependent mitophagy is mediated by the PTEN-induced putative kinase 1 (PINK1) and the E3-ubiquitin ligase PRKN. Under physiological conditions, PINK1 translocated to the inner mitochondrial membrane is cleaved by presenilin-associated rhomboid-like protein, which subsequently leads to the degradation of N-terminal truncated PINK1. However, the loss of mitochondrial transmembrane potential accumulates uncleaved PINK1 by disrupting PINK1 translocation. Accumulated PINK1 recruits and activates PRKN to amplify mitophagy signaling in the cytoplasm, ultimately causing mitophagy [26]. On the other hand, PRKN-independent mitophagy mainly relies on receptor proteins, including BNIP3 (BCL2 interacting protein 3), NIX (NIP3-like protein X), and FUNDC1 (FUN14 domain-containing protein 1), which directly interact with LC3 and GABARAP through their LIR motifs, leading to mitochondrial elimination [27]. By disrupting either of these two major pathways, inefficient mitophagy has been shown to induce the accumulation of dysfunctional mitochondria and contribute to the occurrence and progression of mitochondria-related diseases [28]. Recent studies have shown that abnormal mitophagy also plays a key role in bone diseases [12], which mainly include OA, IVDD, OP, and OS.

## 3. Mitophagy and Osteoarthritis

**Figure 1:**
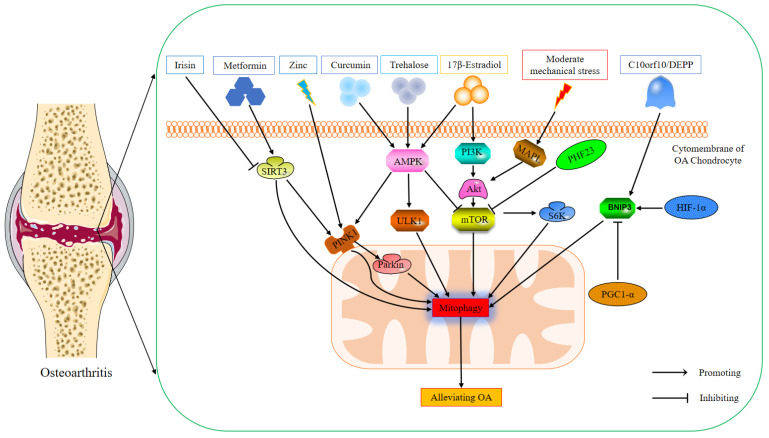
The mechanism of mitophagy in osteoarthritis.

OA is a common osteoarticular disease characterized by cartilage degeneration, subchondral bone sclerosis, synovial inflammation, and lesions of other periarticular structures such as ligaments and menisci. Patients with OA often experience joint pain and joint dysfunction, and even disability [29]. Although OA is a complex process involving the entire joint pathology, progressive degeneration of articular cartilage is recognized as one of the core features of OA pathogenesis [30]. The mitochondrial oxidative phosphorylation in chondrocytes is the main source of their ATP [31]. Studies have found that changes in mitochondrial morphology and function are closely related to the pathogenesis of OA [32]. Therefore, mitochondrial homeostasis is critical for chondrocyte survival and function. Mitophagy is a selective autophagic process involving the removal of damaged mitochondria and the maintenance of mitochondrial homeostasis, resulting in the homeostatic regulation of OA-related gene expression in chondrocytes [33]. Studies have shown that mitophagy is related to OA [14]. Studies have found that spermidine rescues effective autophagic flux and promotes mitophagy by stimulating the expression of autophagy markers to counteract oxidative stress, thereby achieving chondroprotective effects [34]. In addition, another study showed that increasing mitophagy and stabilizing mitochondrial biogenesis could alleviate obesity-induced OA through adenosine A2A receptor therapy [35]. Currently, multiple mitophagy signaling pathways have been identified to be involved in the pathogenesis of OA, including adenosine monophosphate-activated protein kinase (AMPK) [36], Bcl2/adenovirus EIB 19Kd-interacting protein 3 (BNIP3) [37], kinase phosphatase and tensin homolog (PTEN)-induced putative kinase protein 1 (PINK1) [38], and mammalian target of rapamycin (mTOR) [39] (as shown in Figure 1). These findings will contribute to understanding the role of mitophagy in OA.

### 3.1. AMPK Signaling Mediates the Regulation of Mitophagy in Osteoarthritis

AMPK is a major metabolic energy receptor. When the cellular energy charge decreases (the AMP/ATP ratio increases), AMPK is activated to regulate the homeostasis of energy metabolism [40,41]. Given its critical role in the control of energy homeostasis, AMPK has attracted widespread interest as a therapeutic target for potential metabolic diseases, including diabetes, obesity, tumors, and especially osteoarthritis. Tang et al. [42] explored the potential protective effect of trehalose on tert-Butyl hydroperoxide (TBHP)-treated mouse chondrocytes and the surgical destabilization of the medial meniscus (DMM) model of OA. It was found that trehalose exerts an anti-apoptotic effect on chondrocytes by targeting the activation of mitophagy and reticulophagy and restoring the interruption of autophagic flux, thereby blocking the progression of OA, which is related to the activation of AMPK-unc-51 like autophagy activating kinase 1 (ULK1) signaling pathway, suggesting the potential of trehalose in the prevention and treatment of OA. Furthermore, although estrogen (17β-estradiol) has a potential protective effect on OA [43], the underlying mechanism of 17β-estradiol (17β-E2) in OA is unclear. A recent study found that 17β-E2 induced up-regulation of mitophagy through silencing information regulator 2 related enzyme 1 (sirtuin1, SIRT1)-mediated AMPK/mTOR signaling pathway to promote chondrocyte proliferation and viability in vitro [36]. It provides a new perspective on the mechanism of action of 17β-E2 in OA and suggests that the SIRT1-mediated AMPK/mTOR signaling pathway is a potential therapeutic target for OA. In addition, Jin et al. [44] found that curcumin may activate mitophagy through the AMPK/PINK1/Parkin pathway to exert chondroprotective effects in vivo and in vitro.

Overall, AMPK is an important pathway regulating cellular energy and metabolic homeostasis, which may be closely related to the occurrence of mitophagy in OA. Therefore, key molecules on the AMPK signaling pathway may be potential targets for the treatment of OA by interfering with the mitophagy pathway. Although some studies have found that mitophagy-modulating drugs are effective on OA chondrocytes in vitro, it is not enough to prove the actual clinical value due to the huge difference between in vitro and in vivo drug concentrations. It should be noted that there may be differences in the therapeutic effect of different administration methods, such as oral, local subcutaneous, and intra-articular injection. Furthermore, endoplasmic reticulum stress is a key regulatory process in the process of OA pathogenesis and apoptosis [45]. The current study found that trehalose can activate both mitophagy and reticulophagy. The specific mechanism of action can be further explored to provide a reference for the development of new OA drugs.

### 3.2. BNIP3 Signaling Mediates the Regulation of Mitophagy in Osteoarthritis

BNIP3, a well-known autophagy regulator and pro-apoptotic factor, acts as a direct target of transcriptional repression to regulate autophagy through pRB/E2F [46]. BNIP3 is required for hypoxia-induced autophagy in mouse embryonic fibroblasts (MEFs) and multiple tumor cell lines, while the knockdown of BNIP3 exacerbates cell death during hypoxia [46,47]. Lu et al. [14] investigated the effect of hypoxia-inducible factor-1α (HIF-1α) on autophagy in interleukin-1β (IL-1β)-induced human chondrocytes. The study found that with the silencing of hypoxia-inducible factor-1α (HIF-1α), the mitophagy proteins PINK1/Parkin and BNIP3/BAX in chondrocytes were significantly activated, indicating that the mitophagy pathway was activated, thereby initiating the Caspase/Cleaved Caspase 3 cascade of apoptosis, suggesting that abnormal HIF-1α expression leads to the activation of mitophagy-mediated apoptosis. Further, Hu et al. [48] verified that HIF-1α plays an important role in enhancing extracellular matrix (ECM) synthesis at the transcriptional level and inhibiting hypoxia-induced ECM degradation by enhancing the HIF-1α/BNIP3 signaling pathway of mitophagy in vivo and in vitro. The study also found a dose-dependent increase in BNIP3 expression following prolylhydroxylase (PHD) inhibitor dimethyloxalylglycine (DMOG)-induced HIF-1α expression. In addition, another study found that the expression of peroxisome proliferator-activated receptor-gamma co-activator 1-alpha (PGC1α) was significantly reduced in OA, whereas the knockdown of PGC1α activated the parkin RBR E3 ubiquitin protein ligase (PRKN)-independent selective mitophagy pathway by upregulating BCL2 and BNIP3 [49]. This study also demonstrated that overexpression of BNIP3 in immature mice articular chondrocytes (iMAC) results in defective mitochondrial depolarization, suggesting that mitochondrial depolarization promoted by BNIP3 can sufficiently induce mitophagy, leading to an imbalance in cartilage matrix homeostasis. Therefore, modulation of the PGC1α/BNIP3 mitophagy axis may have therapeutic effects on cartilage degradation in OA. Moreover, Kuwahara et al. [37] explored the effect of decimal protein induced by progesterone (C10orf10/DEPP) on chondrocyte mitophagy in vivo and in vitro. They found that knockout DEPP mice exhibited severe cartilage degeneration due to mitochondrial autophagy dysfunction and chondrocyte death in a mouse model of OA. As a major stress-inducible gene in OA, DPEE actively promotes mitophagy and maintains chondrocyte viability through BNIP3 signaling in response to stress.

In summary, BNIP3-mediated mitophagy can protect or destroy chondrocytes during the pathogenesis of OA. The detailed regulatory mechanism of the interaction between PGC-1α and BNIP3 in mitochondria is unclear. Furthermore, HIF-1α-mediated mitophagy played a role in suppressing apoptosis and senescence in hypoxia-stimulated chondrocytes. However, the underlying mechanisms involved remain unclear. Moreover, mitochondrial protective effects of HIF-1α through inducing transcription of anti-aging and anti-apoptotic genes also need to be further explored. Overall, the mechanism of mitophagy that reduces chondrocyte senescence and apoptosis under a hypoxic environment requires further study.

### 3.3. PINK1 Signaling Mediates the Regulation of Mitophagy in Osteoarthritis

PINK1 is a serine/threonine protein kinase with a mitochondrial target sequence that is processed by mitochondrial proteases on the mitochondrial membrane of healthy mitochondria. The main function of PINK1 is to degrade the proteasome [50]. During the initial stage of mitophagy, defective mitochondria are engulfed by autophagosomes through PINK1 recognition and subsequently fused with lysosomes for hydrolytic degradation [51]. The presence of PINK1 on the mitochondrial surface is enhanced by mitochondrial damage and depolarization, which prevents the mitochondrial import of Pink1 [52]. Previous studies have shown that PINK1-mediated mitophagy has a positive role in the treatment of RA [53]. Some scholars have also found a similar role in OA. Shin et al. [38] explored the role of PINK1-mediated mitophagy in mitochondrial fission in a monosodium iodoacetate (MIA)-induced model of OA, which is a key process in OA pathogenesis. The results show that PINK1-mediated mitophagy may be responsible for chondrocyte death after MIA treatment, while PINK1 deficiency is associated with MIA-induced reduction of cartilage damage and pain, suggesting that targeting the PINK1 pathway may be a potential therapeutic strategy for OA. Interestingly, Huang et al. [54] found that zinc treatment could counteract cartilage damage induced by MIA treatment by reversing low levels of ATP and upregulating the PINK1-dependent selective mitophagy pathway. In addition, another study found that irisin increases mitochondrial membrane potential, ATP production, and catalase by reversing the expression of signaling molecules, including SIRT3, to attenuate IL-1β-mediated mitophagy and autophagosome formation in chondrocytes [55] which suggests that SIRT3 signaling may also be involved in the regulation of mitophagy. Furthermore, Wang et al. [56] reported the inhibitory properties of metformin on mitochondrial damage in vitro. This study found that metformin partially reversed the enhanced catabolism and ROS induced by IL-1β through mitophagy associated with activation of the SIRT3/PINK1/Parkin signaling pathway. The above results further illustrate that mitophagy may be a therapeutic target for OA.

In general, PINK1-mediated mitophagy may be an effective strategy for OA treatment in the context of mitochondrial damage. Although PINK1 is a well-established mitochondrial-targeting protein, its expression is highly heterogeneous. PINK1 has been reported to be associated with the inner mitochondrial membrane, the mitochondrial intermembrane space, the outer mitochondrial membrane, and even the cytoplasm [38]. Therefore, further understanding of the pathophysiological link between mitophagy-induced cartilage damage and PINK1 expression is necessary for targeted therapy of OA. Notably, a novel function of PTEN-long (PTEN-L) has been reported as a ubiquitin protein phosphatase that counteracts PINK1-mediated ubiquitin phosphorylation, thereby blocking the mitophagy-induced feed-forward mechanism and ultimately inhibiting mitophagy [57]. Therefore, an in-depth understanding of this novel function of PTEN-L may provide help for the application of PINK1-mediated mitophagy in OA treatment.

### 3.4. mTOR Signaling Mediates the Regulation of Mitophagy in Osteoarthritis

mTOR, a serine/threonine protein kinase, is a key regulator of cell growth, metabolism, and survival [58]. Hypoxia, ROS, and deprivation of nutrients and energy are all factors that inhibit mTOR [59]. It has been reported that the decreased expression of autophagy-related proteins in OA cartilage is related to the overexpression of mTOR. The regulation of Unc-51–like kinase 1 (ULK1)/AMPK signaling pathway by mTOR may be involved in regulating autophagy signaling and the balance between catabolic and anabolic factors in articular cartilage [60]. Therefore, it is speculated that mTOR-mediated mitophagy is involved in the regulation of OA. Plant homeodomain finger protein 23 (PHF23) is a novel autophagy inhibitory gene that inhibits autophagy and accelerates apoptosis in OA chondrocytes [61]. Some scholars established stable chondrocyte cell lines with PHF23 overexpression or knockdown through PHF23-specific lentiviral vectors to explore the anti-autophagy mechanism of PHF23 [62]. They found that PHF23 downregulated IL-1β-induced mitophagy in chondrocytes. Furthermore, the knockdown of PHF23 promoted mitophagy by enhancing the activation of the AMPK pathway and attenuating the activity of the mTOR/S6K pathway. However, further research is needed to provide more evidence. In addition, Zhang et al. [39] found that mechanical stress promotes chondrocyte mitophagy by regulating the MAPL/Akt/mTOR signaling pathway in an intensity-dependent manner. However, excessive mechanical stress can induce uncoupling of fission and fusion to disrupt the balance of mitochondrial dynamics and mitochondrial homeostasis, leading to chondrocyte apoptosis. He et al. [63] further found that cartilage degradation caused by mechanical overload is related to the impairment of mitophagy. Urolithin A (UA) treatment partially restores mitophagy in mechanically damaged chondrocytes. Cumulative evidence shows that mitophagy is a “double-edged sword”: moderate mitophagy can protect cells from apoptosis, while excessive mitophagy can degrade the entire cytoplasm, leading to cell death [4]. Fan et al. [4] revealed that 17β-E2 inhibits mitophagy in ATDC5 chondrocytes through G-protein coupled estrogen receptor (GPER/GPR30) and PI3K/Akt/mTOR signaling pathways, suggesting that PI3K/Akt/mTOR signaling may be a potential target for mitochondrial damage-related OA therapy.

Although the relationship between mTOR signaling and mitophagy has been initially confirmed in the occurrence and development of OA, further exploration of the specific mechanisms in vivo and in vitro is required to determine the clinical therapeutic potential of mTOR signaling-mediated mitophagy in OA. Furthermore, increasing evidence suggests that inhibition of osteoclast activity can alter subchondral bone remodeling and alleviate articular cartilage degeneration due to abnormal activation of subchondral bone osteoclasts mediating the progression of OA [64]. Multiple autophagy-related genes, such as TNFSF11 and ATG5, have been found to affect the differentiation and function of osteoclasts [65]. Moreover, autophagy increases osteoclast activity and stimulates osteoclast-mediated bone resorption in vivo and in vitro [66]. However, current research on the roles of osteoclast autophagy and mitophagy in the development of OA is limited [67]. Exploring the osteoclast mitophagy regulatory network may provide new ideas for elucidating the specific mechanism of OA.

## 4. Mitophagy and Intervertebral Disc Degeneration

IVDD can cause instability, deformity, and narrowing of the spinal segment, which is one of the main causes of physical disability and low back pain [68]. The main pathological features of IVDD are ECM degradation, senescence and apoptosis of nucleus pulposus cells (NPCs), and impaired self-repair function [69]. As primary cells residing in the nucleus pulposus (NP), NPCs are responsible for the synthesis of ECM and maintain the biological and mechanical properties of the intervertebral disc [70]. The dysfunction of NP tissue plays a crucial role in the pathological development of IVDD [71]. The intervertebral disc is the largest avascular tissue in mammals. Therefore, NPCs mainly rely on the mitochondrial glycolytic pathway for energy supply [28]. When mitochondria are damaged, or dysfunctional mitochondria are excessively accumulated, multiple stress signals are generated, which in turn lead to NPCs dysfunction and eventual programmed cell death [72]. Therefore, maintaining normal mitochondrial function is critical for NPCs homeostasis. Mitophagy is a major mechanism of mitochondrial quality control [27]. Abnormal mitophagy can cause mitochondrial dysfunction and death of NPCs, eventually leading to tissue damage and disease, such as IVDD [73]. Previous studies have found that the anti-inflammatory molecule A20 can alleviate the pyroptosis and apoptosis of NPCs by promoting mitophagy and stabilizing mitochondrial dynamics [74]. Another study found that the knockdown of the transcription factor forkhead box O3 (FOXO3) could inhibit mitophagy in NPCs under nutrient-deprived conditions, thereby maintaining the ECM composition in NPCs [75]. Therefore, rational induction and regulation of the mitophagy pathway in NPCs may play an important role in the prevention and treatment of IVDD, which may involve signaling pathways including sirtuins (SIRTs), Parkin, and HIF-1α (as shown in Table 1).

### 4.1. SIRTs Signaling Mediates the Regulation of Mitophagy in Intervertebral Disc Degeneration

The SIRTs are a family of nicotinamide adenine dinucleotide (NAD)-dependent histone deacetylases (HDACs) [93]. Recent studies have reported that SIRTs are involved in various cellular physiological processes, such as histone deacetylation, protein acylation, and deacetylation [94], as well as various antioxidant and oxidative stress-related-processes and functions, such as metabolism, DNA damage repair, mitochondrial function and life [95]. Some members of the SIRTs family, such as SIRT1, SIRT2, and SIRT3, have also been found to play key regulatory roles in IVDD progression [96,97,98]. The role of mitophagy in IVDD has been demonstrated [27]. The underlying mechanisms of SIRTs family members in mitophagy-mediated IVDD are not fully understood. SIRT1 is a nicotinamide-dependent class 3 histone deacetylase involved in the regulation of multiple cellular processes, such as cell cycle, metabolism, and senescence, which are critical for cell survival [99,100]. Given the antioxidant and mitophagy-related properties of SIRT1 [101], Wang et al. [77] found that SIRT1 could alleviate mechanical stress-induced senescence in human NP cells under high-intensity compression by activating mitophagy. In addition to anti-aging effects, SIRT1 also plays a key role in mitophagy and apoptosis in aging-related diseases. Xie et al. [102] found that circular RNA ERCC2 (circERCC2) could inhibit tert-butyl hydroperoxide (TBHP)-induced apoptosis, ECM degradation, and activated mitophagy in NPCs by directly targeting the miR-182-5p/SIRT1 signaling axis, ultimately alleviating IVDD [76]. Furthermore, as an important member of the Sirtuins family, SIRT2 plays a crucial role in protecting cells from ROS [19]. It has been reported that SIRT2 inhibits mitophagy by acting on peroxisome proliferator-activated receptor gamma coactivator 1-alpha (PGC-1α) to protect annulus fibrosus (AF) cells from oxidative stress-induced apoptosis [19]. In contrast, SIRT3 maintains mitochondrial homeostasis by promoting mitochondrial dynamics, antioxidation, mitophagy, and deacetylation of manganese superoxide dismutase (MnSOD), thereby enhancing its biological activity in aging NPCs, suggesting that SIRT3 may play an important role in the mitochondrial function of NPCs against oxidative stress [79]. Hu et al. [78] reported that activation of the Nuclear respiratory factor 2 (Nrf2)/SIRT3 signaling axis could mediate mitophagy to inhibit NP apoptosis and prevent IVDD. In addition, previous studies have found that adenosine monophosphate-activated protein kinase (AMPK) signaling is involved in mitophagy-mediated effects to inhibit NP cell apoptosis, thereby alleviating IVDD [80]. Honokiol (HKL) is a small molecular weight natural compound with analgesic and anti-inflammatory properties extracted from Magnolia bark, which can enhance mitochondrial antioxidant, mitochondrial dynamics, and mitophagy through the AMPK/PGC-1α/SIRT3 signaling pathway, thereby partially reversing oxidative stress-induced apoptosis and senescence in NPCs, and ultimately improving mitochondrial dysfunction [79]. The above studies suggest that SIRT3 may be a potential therapeutic target for IVDD.

In summary, SIRT1, SIRT2, and SIRT3 are involved in the occurrence and development of IVDD by regulating mitophagy. The difference is that SIRT1 and SIRT3 play an active therapeutic role by promoting mitophagy, while SIRT2 plays a similar therapeutic role by inhibiting mitophagy. In addition, SIRT4 and SIRT5 in the Sirtuins family have also been reported to be involved in the regulation of mitochondria-related activities [79]. Another study found that SIRT6 prevented extracellular matrix degradation in the nucleus pulposus in IVDD by inhibiting the nuclear factor-κB (NF-κB) pathway [103]. Therefore, it is speculated that other members of the Sirtuins family may also be involved in mitophagy activity in IVDD. Notably, mitophagy is a rather complex and controversial process in regulating cell survival. There may be more than one mediator in the regulation of mitophagy by the Sirtuins family. The pathological mechanism of the involvement of Sirtuins family-induced mitophagy in NP degradation remains to be further studied.

### 4.2. PINK1/Parkin Signaling Mediates the Regulation of Mitophagy in Intervertebral Disc Degeneration

PINK1 can phosphorylate Parkin cooperating with PTEN-induced kinase 1 (PINK1) to convert it into an active phospho-ubiquitin-dependent E3 ligase to abrogate the loss of damaged mitochondria in response to MMPs [104]. Parkin-mediated mitophagy is a widely studied mitophagy pathway. The mechanism is that Parkin is translocated to defective mitochondria, and p62/SQSTM1 is recruited, followed by autophagosome phagocytosis of damaged mitochondria and lysosomal degradation [24]. As a protective protein, Parkin plays an active role in preventing degenerative diseases such as Parkinson’s disease [105], osteoarthritis [106], and Alzheimer’s disease [107]. Furthermore, a recent study found that mitochondrial-targeted antioxidant mitoquinone (MitoQ) ameliorated mitochondrial dysfunction and redox imbalance by promoting PINK1/Parkin-mediated mitophagy and restoring mitophagy flux, thereby preventing IVDD [83]. This study lays the foundation for further investigation of the role of Parkin-mediated mitophagy in IVDD. It has been found that Parkin signaling plays a regulatory role in IVDD through mitochondrial autophagy mediated by Parkin alone or PINK1/Parkin signaling pathway.

Parkin signaling plays a key role in mitophagy induction, which regulates the ubiquitination of mitochondrial outer membrane proteins and promotes dysfunctional mitochondrial degradation [87]. In addition, a meta-analysis of 4600 people by Williams et al. [108] found CpG island methylation of the PARK2 gene (encode Parkin protein) promoter in IVDD patients, indicating that Parkin signaling is closely related to IVDD. Lan et al. [87] reported that activation of Parkin could prevent oxidative stress-induced apoptosis and mitochondrial dysfunction in rat NPCs by promoting mitophagy, suggesting that Parkin is a promising target for IVDD therapy. Some scholars further found that knockdown of leucine-rich repeat kinase 2 (LRRK2) can promote Parkin recruitment and activate Parkin-mediated mitophagy to inhibit mitochondria-dependent apoptosis of NPCs induced by oxidative stress, thereby delaying the development of IVDD [88]. Melatonin, which is also essentially a protein, was also found to have the same effect on the development of IVDD [89]. Melatonin treatment induces Parkin-mediated mitophagy in NPCs to clear damaged mitochondria and reduce the release of ROS and apoptotic factors, thereby inhibiting NPCs apoptosis and ECM degeneration induced by oxidative stress. Furthermore, traditional Chinese medicine has also been found to improve IVDD through this pathway. Salidroside (Sal), extracted from the traditional Chinese medicine Rhodiola, ameliorated mitochondrial damage and apoptosis in NPCs through Parkin-mediated mitophagy activation [91]. In addition, the cartilage endplate (CEP) is the main pathway for nutrient uptake and waste excretion in the intervertebral disc (IVD) and is also important for maintaining IVD homeostasis. Studies have shown that endplate chondrocyte apoptosis can lead to CEP degeneration and the initiation of IVDD [109]. Kang et al. [90] found that Parkin-mediated mitophagy and Nrf2-mediated antioxidant system prevented oxidative stress-induced apoptosis of intervertebral endplate chondrocytes and contributed to endplate chondrocyte survival. Therefore, further studies on endplate chondrocyte apoptosis are needed, which may provide potential therapeutic strategies for IVDD.

Among the specific regulators of mitophagy, the PINK1-Parkin signaling pathway is one of the main pathways that have been discovered to regulate mitophagy [110]. PINK1 is a protein kinase that aggregates at the outer mitochondrial membrane (OMM) in response to the reduction of MMPs caused by damage/dysfunction. PINK1 clustered in OMM recruits Parkin from the cytoplasm to the OMM, as its E3 activity promotes mitophagy through ubiquitination of mitochondrial proteins, leading to mitochondrial degradation [111]. Wang et al. [81] found that PINK1 is involved in protective resistance to oxidative stress-induced senescence through PINK1/Parkin-mediated mitophagy in human degenerative NPCs. In another experiment, Huang et al. [86] revealed that excessive degradation of mitochondria in NPCs by mitophagy may accelerate the senescence of NPCs under continuous mechanical compressive stress, indicating that modulating PINK1/Parkin-mediated mitophagy may be a potential therapy for IVDD. Furthermore, a study on NPCs senescence in IVDD found that macrophage migration inhibitory factor (MIF) deficiency aggravated ROS accumulation, mitochondrial dysfunction, and NPCs senescence under overloaded mechanical compression, which may be related to the regulation of PINK1/Parkin-mediated mitophagy by MIF [85]. In addition to being involved in the regulation of NPCs senescence, PINK1/Parkin signaling is also involved in the death of NPCs. Chen et al. [84] found that inhibition of mitochondrial outer membrane protein mitofusin2 (Mfn2) in NP tissues may be a determinant of aggravated autophagy damage during IVDD, leading to mitochondrial dysfunction and death of NPCs. Further studies found that overexpression of Mfn2 restores blocked autophagic flux and promotes ROS-dependent mitophagy via the PINK1/Parkin signaling pathway. In addition, the PINK1/Parkin signaling pathway was also found to be involved in the regulation of pyroptosis in NPCs. Ma et al. [82] reported that SIRT1 could ameliorate IL-1β-induced pyroptosis in NPCs and reduce mitochondrial ROS production to alleviate mitochondrial dysfunction through PINK1/Parkin-mediated mitophagy. The study also found that IL-1β stimulated NLRP3 inflammasome activation through mitochondrial oxidative stress injury and mitochondrial ROS production. Therefore, regulating NLRP3 inflammasome and mitophagy may be a potential therapeutic strategy for inflammation-related IVDD.

Overall, most studies support a positive effect of Parkin-mediated mitophagy on remission of IVDD, while a few studies show a negative effect of Parkin-mediated mitophagy on IVDD remission, suggesting that precise regulation of the extent of mitophagy may be critical in the treatment of IVDD. In addition, the metabolism of ECM is a major contributor to IVDD [88]. ECM metabolism can be assessed in the context of Parkin-mediated mitophagy. Furthermore, studies have found that inhibition of Nrf2 can increase H2O2-induced Parkin expression and LC3-II/I ratio. Inhibition of Parkin also increases H2O2-induced Nrf2 expression in endplate chondrocytes [90], suggesting that Parkin and Nrf2 may interact in ROS-excessive endplate chondrocytes. Further studies are required to fully elucidate the regulatory mechanisms of the interaction between Parkin and Nrf2 signaling, thereby contributing to the development of effective therapeutic strategies to inhibit the progression of IVDD.

### 4.3. HIF-1α Signaling Mediates the Regulation of Mitophagy in Intervertebral Disc Degeneration

Healthy intervertebral disc tissue, the largest avascular tissue in mammals, survives the physiological hypoxic niche through the robust expression of HIF-1α. HIF-1α, a master transcription factor stably expressed in NPs, adapts NP cells to hypoxia by upregulating glycolysis [112]. Under hypoxic conditions, HIF-1α coordinates glycolysis and tricarboxylic acid (TCA) cycle interactions and restricts oxidative phosphorylation in NP cells. Loss of HIF-1α in hypoxia probably leads to cell death through a notable reduction in glycolytic metabolism and dysregulated TCA flux implying altered mitochondrial activity [28]. Therefore, HIF-1α is critical for the homeostasis and survival of NP cells under hypoxia. Madhu et al. [28] found that hypoxia promotes mitochondrial fission through HIF-1α and triggers mitophagy to maintain NPCs homeostasis by controlling the translocation of BNIP3 to mitochondria. They also found that sequestration of BNIP3 in HIF-1α-silenced cells was compensated for by NIX and/or PINK1-PARK2 in HIF-1α-silenced NPCs [28]. It suggests that hypoxic regulation of mitochondrial metabolism and mitophagy is dependent on HIF-1α-BNIP3 signaling in NP cells. Furthermore, studies have shown that proper mitophagy activation is protective against apoptosis, whereas excessive mitophagy leads to apoptosis [113]. Xu et al. [92] found that oxidative stress-induced NP apoptosis and mitophagy activation were regulated by HIF-1α/NADH dehydrogenase (ubiquinone) 1 alpha subcomplex subunit 4-like 2 gene (NDUFA4L2) signaling, while the mitophagy inhibitor cyclosporin A protected NPCs from oxidative stress and apoptosis. Further experiments showed that up-regulation of NDUFA4L2 ameliorated the apoptosis of NPCs by inhibiting excessive mitophagy, ultimately alleviating IVDD [92]. The above studies demonstrate that HIF-1α-mediated mitophagy may be beneficial or detrimental in IVDD. Further studies are needed to reveal the regulatory role of HIF-1α-mediated mitophagy in IVDD, thus rationally regulating the expression of HIF-1α to play more beneficial roles at different stages of IVDD development.

## 5. Mitophagy and Osteoporosis

**Figure 2:**
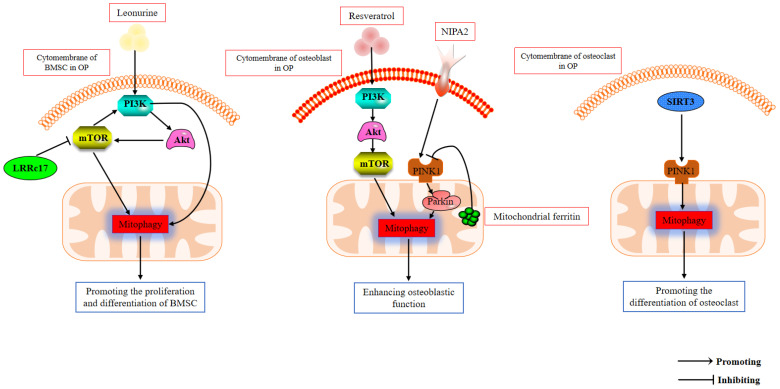
The mechanism of mitophagy in osteoporosis.

OP is a systemic skeletal disease characterized by decreased bone mineral density (BMD), alterations in bone microarchitecture, and increased bone fragility, with a predilection for the elderly [65]. In addition to age-related OP, other factors such as inappropriate glucocorticoid use, estrogen deficiency, diabetes, and malnutrition are common causes of secondary OP. Recently, pharmacological options aim at either osteoclastogenesis inhibition or osteoblastogenesis stimulation. However, currently, the clinical treatment effect of OP is not ideal. Osteoclast-mediated bone breakdown may occur, leading to impaired therapeutic outcomes [114]. Therefore, more effective osteoporosis treatment strategies are urgently needed. Mitophagy not only protects cells from apoptosis by scavenging damaged mitochondria caused by oxidative stress but also promotes the biosynthesis of new mitochondria, which is critical for cellular homeostasis [115]. There is increasing evidence that mitophagy is closely related to aging and age-related diseases [116]. Although mitophagy is a physiological activity under pathological or specific conditions, mitophagy can also be altered to promote aging-related diseases [117]. Accumulating evidence suggests that aberrant mitophagy plays a key role in stem cell differentiation and in bone metabolism disorders [12,118]. Recent studies have found that mitophagy is involved in the regulation of OP [119,120], which may be related to signals such as phosphoinositide 3-kinase (PI3K) [115] and Pink1 [121] (as shown in Figure 2).

### 5.1. PI3K Signaling Mediates the Regulation of Mitophagy in Osteoporosis

PI3K, which belongs to the lipid kinase family, is involved in the formation of eukaryotic cell membranes and regulates cell signal transduction, energy metabolism, cell cycle, and other intracellular processes [122]. During vesicle nucleation, the regulation of autophagy by PI3K as a signaling hub has two aspects. On the one hand, class-I PI3K inhibits autophagy; on the other hand, class-III PI3K promotes autophagy [123,124]. Recent studies have shown that PI3K signaling is also involved in mitophagy [125]. The role of mitophagy, as a special form of autophagy, and PI3K signaling in OP regulation has been preliminarily explored. It has been demonstrated that the senescence of bone marrow mesenchymal stem cells (BMSCs) is associated with age-related diseases, including OP. Furthermore, BMSCs have the potential to differentiate into osteoblasts and promote osteogenesis and angiogenesis through paracrine action, thereby playing a role in alleviating OP progression [126]. Liu et al. [127] found that LRRc17 knockdown activates mitophagy by inhibiting the mTOR/PI3K pathway and reducing mitochondrial dysfunction, thereby rejuvenating senescent BMSCs and ultimately relieving OP. In addition, Leonurine, a natural compound extracted from the traditional Chinese medicine Leonurus, has attracted widespread attention due to its potent antioxidant effect [128]. Zhao et al. [129] showed that Motherwort activates mitophagy by inhibiting the PI3K/Akt/mTOR pathway to prevent the production of ROS in BMSCs and contribute to mitochondrial quality control, thereby protecting the proliferation and differentiation of BMSCs from oxidative stress, suggesting that Motherwort has potential application value in OP. This study provides evidence that Chinese medicinal components promote the osteogenic differentiation of BMSCs through mitophagy. In addition, it is also important to explore whether Chinese medicinal components can regulate the survival or function of osteoblasts through the mitophagy pathway, as osteoblast dysfunction is one of the main causes of bone loss [130]. Yang et al. [115] reported that Resveratrol, a traditional Chinese medicine ingredient, enhanced mitophagy in osteoblasts of OP rats by up-regulating the expression of SIRT1 and activating the PI3K/Akt/mTOR signaling pathway and significantly improved bone mass, and decreased serum alkaline phosphatase and osteocalcin levels in OP rats.

Taken together, inhibition of PI3K signaling can activate mitophagy in BMSCs, thereby enhancing the therapeutic effect of BMSCs on OP, while activation of PI3K signaling can enhance mitophagy in osteoblasts, thereby alleviating OP progression. The PI3K pathway has been shown to be involved in the regulation of cellular endogenous ROS levels and cell fate decisions [131], which is important for cell survival [132]. In addition, some scholars have proposed that the PI3K pathway may be a double-edged sword. The inhibition or promotion of mitophagy by PI3K depends on different microenvironments [133]. Therefore, it is necessary to further clarify the crosstalk relationship between PI3K and its upstream and downstream signaling molecules, which may be the key to explaining the different effects of PI3K on mitophagy in different situations.

### 5.2. PINK1 Mediates the Regulation of Mitophagy in Osteoporosis

PINK1 is a core regulator of mitophagy [134]. Under physiological conditions, PINK1 translocates to the healthy inner mitochondrial membrane, where it is rapidly degraded. When mitochondria are damaged with depolarization or loss of membrane potential, PINK1 accumulates on the mitochondrial outer membrane and phosphorylates ubiquitin and other mitochondrial outer membrane proteins, including Parkin, following recruitment of autophagy machinery, autophagosome formation, and eventual clearance of unhealthy mitochondria [135]. Previous studies have shown that PINK1 is involved in the normal regulation of mitophagy. Disruption of PINK1 leads to impaired mitophagy [136]. Impaired mitochondrial function negatively affects osteoblast differentiation and mineralization [137], while restoration of mitophagy contributes to the mitigation of hormone-induced bone loss [138]. Therefore, it is reasonable to speculate that PINK1-mediated mitophagy is involved in the regulation of OP occurrence and development.

Mitochondrial protein turnover caused by mitophagy is critical for maintaining healthy mitochondrial function [139]. The activity of PINK1 as a sensor of mitochondrial damage is tightly regulated by mitochondria. In response to mitochondrial damage, activated PINK1 normally cooperates with Parkin ubiquitin ligase to promote mitophagy and regulate mitochondrial quality control [140]. Activation of PINK1 is necessary during osteoblast differentiation, which is associated with mitochondrial quality control and low reactive oxygen species production. Lee et al. [121] found that the expression of endogenous PINK1 is increased during osteogenic differentiation. Downregulation of PINK1 impairs mitochondrial homeostasis, increases mitochondrial ROS generation, and inhibits osteoblast differentiation. Further study showed that PINK1 deletion aggravates bone loss in ovariectomized mice. The above results indicate that PINK1 may be involved in the regulation of bone metabolism. Therefore, enhancing PINK1 activity may be a therapeutic target for OP. In addition, type 2 diabetic osteoporosis (T2DOP) is a diabetic complication that seriously affects the quality of life of the elderly. Zhao et al. [141] found that the highly selective magnesium transporter non-imprinted in Prader-Willi/Angelman syndrome region protein 2 (NIPA2) could inactivate PINK1/Parkin-mediated mitophagy through the PGC-1α/FoxO3a/MMP pathway, thereby positively regulating the osteogenic ability of high glucose-treated osteoblasts. In addition, ferroptosis is a cell death pattern closely related to iron metabolism [142]. Iron disorders are representative ionic disorders in diabetic patients. The imbalance of iron homeostasis is one of the factors leading to OP [143]. Wang et al. [144] reported that down-regulation of mitochondrial ferritin (FtMt) could enhance osteoblast mitophagy through the PINK1/Parkin pathway to promote ferroptosis of osteoblasts in T2DOP and inhibit the osteogenic function, suggesting that FtMt may be an effective target for T2DOP therapy. Furthermore, alterations in the mitochondrial activity of osteoblasts and osteoclasts have been associated with the bone loss associated with aging and estrogen deficiency, which are two common causes of osteoporosis [139]. It has been demonstrated that SIRT3-mediated mitophagy in BMSCs plays a protective role in OP development [145]. Ling et al. [139] further revealed that SIRT3-mediated PINK1 deacetylation can promote osteoclast differentiation by regulating osteoclast mitophagy. Knockout of Sirt3 or Sirt3 inhibitor LC-0296 can partially inhibit the formation and function of osteoclasts and attenuate the increase in bone resorption and loss of bone mass caused by estrogen deficiency or aging, suggesting that targeted inhibition of Sirt3 could impair osteoclast mitochondrial function through PINK1-mediated mitophagy and prevent OP caused by aging and estrogen deficiency. Nonetheless, further analysis is required. For example, proteomics can be used to identify specific target proteins and mitochondrial regulatory pathways that SIRT3 mediates during osteoclast development.

In summary, the above studies suggest that the PINK1-mediated mitophagy pathway can not only promote the differentiation and function of osteoblasts but also inhibit the formation and/or activity of osteoclasts, thereby alleviating the progression of OP. Ferroptosis, a new form of cell death discovered in recent years, is involved in osteoporotic bone remodeling and mainly affects the interaction between bone formation and bone resorption. Targeted regulation of ferroptosis can effectively alleviate OP [146]. Although studies have found that mitophagy can induce ferroptosis [144], the specific mechanism of mitophagy-induced ferroptosis remains unclear. Therefore, the mechanism of mitophagy in ferroptosis needs to be further studied to provide new ideas for the treatment of OP.

## 6. Mitophagy and Osteosarcoma

OS is a common malignant bone tumor that occurs in children and young adults with the characteristics of easy recurrence, strong invasiveness, and early metastasis [147,148]. Complications such as a physical disability may occur in conventional surgical treatment, resulting in a poor prognosis. In recent years, neoadjuvant chemotherapies have become a common strategy for OS. However, there are also disadvantages of drug resistance and large side effects [149]. Despite the revolutionary progress of neoadjuvant chemotherapy combined with aggressive surgical resection, the clinical outcomes of patients with OS have not been significantly improved [150]. Therefore, there is an urgent need to discover new effective therapeutic targets to develop more effective OS treatment strategies. Autophagy is a self-protective mechanism in cells. Dysregulated autophagy is involved in a variety of pathological processes, including cancer [151]. Furthermore, since mitochondria are frequently altered in tumor cells (Warbung effect), cancer metabolism is highly dependent on glycolysis [152]. Mitochondria-dependent apoptosis is counterbalanced by their selective degradation via autophagy (a process known as mitophagy) [153]. Mitophagy can promote tumor progression and have cytoprotective effects from different anticancer agents [154,155]. Previous studies have found that the antitumor drug Norcantharidin inhibits proliferation and promotes apoptosis in human OS cells involved in mitophagy [150], while the anti-inflammatory phytochemical parthenolide-induced OS cell death can be attributed to mitophagy [156]. The above results suggest that mitophagy is involved in the survival regulation of OS cells, which is mainly related to BNIP3 signaling [157].

BNIP3 is a member of the proapoptotic Bcl-2 subfamily located in the OMM [158]. BNIP3 can inhibit the function of the anti-apoptotic protein Bcl-2 and trigger the mitophagy pathway [159]. BNIP3 targets mitochondria through distinct clearance pathways regulated by distinct transcription factors such as hypoxia and ubiquitination [47]. Furthermore, there is growing evidence that high expression levels of BNIP3 are associated with aggressive cancer behavior in different tumor types, such as breast, colorectal, prostate, and endometrium [160]. These studies provide ample evidence for the regulatory role of BNIP3-mediated mitophagy in different types of cancer. ZnO nanoparticles (NPs) are a potential cancer nanomedicine. Li et al. [157,161] revealed that NP-induced OS cell killing follows a HIF-1α-BNIP3-LC3B-mediated mitophagy pathway. Further studies found that ZnO NPs entered human OS cells through the endocytic pathway and destroyed mitochondria before being captured in lysosomes, thereby initiating mitophagy-Zn^2+-^ROS-mitochondrial axis-mediated OS cell apoptosis. It is worth noting that there are many challenges in the treatment of OS, especially the low survival rate after metastasis. It is crucial to explore therapeutic strategies that can effectively inhibit OS cell metastasis. It was found that Zn^2+^ released from ZnO NPs induced the degradation of β-catenin through the HIF-1α/BNIP3/LC3B-mediated mitophagy degradation pathway, thereby activating the HIF-1 and Wnt pathways to inhibit OS cell metastasis [162]. In addition, drug resistance is also an important reason that hinders the further improvement of the 5-year survival rate of OS patients [163]. It has been demonstrated that cancer cells can evade chemotherapy with the help of mitophagy. Enhanced mitophagy in cancer cells may contribute to chemoresistance through increased mitochondrial clearance, facilitating the removal of the source of cytochrome c [164,165]. Therefore, the regulation of mitophagy may be a strategy to deal with OS resistance. Vianello et al. [160] found higher expression of the mitophagic factor BNIP3 levels and enhanced mitophagy in both in vitro cisplatin (CDDP) resistant models and cancer patients’ samples resistant to platinum-based chemotherapy. Further studies found that BNIP3 ablation re-sensitizes resistant clones to CDDP by inhibiting mitophagy, suggesting that BNIP3 may be a potential target to overcome the phenomenon of OS treatment resistance.

Taken together, effective regulation of the BNIP3-mediated mitophagy pathway may be one of the effective strategies to kill OS cells, inhibit OS cell metastasis, and reduce OS drug resistance. Interestingly, the first two effects (killing OS cells and inhibiting OS cell metastasis) depend on the activation of BNIP3-mediated mitophagy, whereas inhibition of BNIP3-mediated mitophagy contributes to the reduction of OS resistance. In addition, some scholars found that during chemotherapy, OS cell death may be related to PINK1/Parkin-mediated mitophagy [156], suggesting that there may be other mitophagy-related signaling pathways in OS.

## 7. Conclusions and Perspectives

Bones are in the process of dynamic change throughout life. Osteoblasts, osteoclasts, osteocytes, chondrocytes, and bone marrow mesenchymal stem cells contained in bone are involved in the pathogenesis of bone-related diseases. As the main energy supply site of these cells, mitochondrial quality control is extremely important. Mitophagy, as the main mechanism of mitochondrial quality control, plays a role in maintaining cellular homeostasis. By reviewing the literature on mitophagy and bone-related diseases, this paper found that the signaling pathways involved in the regulation of mitophagy in bone metabolic diseases mainly include: PINK1/Parkin, BNIP3, AMPK, mTOR, SIRT1, SIRT2, SIRT3, HIF-1α, and PI3K, of which PINK1/Parkin is the most widely studied signal pathway for mitophagy regulation. However, there may be other signaling pathways associated with mitophagy that require further investigation in bone-related diseases. In addition, some drugs such as curcumin, melatonin, and leonurine have been found to have the potential to treat multiple bone-related diseases by modulating the level of mitophagy, which show a positive therapeutic effect. However, different administration methods such as oral, local subcutaneous, and intraarticular injection still need to be further explored to ensure the best efficacy, as well as the optimal administration concentration and dosage.

Although the regulation of mitophagy has shown great potential in the treatment of bone diseases, there are still a series of issues to be further clarified: (1) the characteristics of different stages of drug-activated mitophagy need to be further elucidated, including labeling damaged mitochondria, autophagosome formation, lysosome and autophagosome fusion, and exocytosis; (2) although many signaling pathways have been found to be involved in the mediation of mitophagy, the precise regulatory mechanism needs further research to discover specific effective drug targets; (3) many studies are limited by observing mitophagy in cells at only one time point. More experimental validation with different time points or a long time after regulation of mitophagy should be performed to identify potential biomarkers, targets, or long-term effects; (4) the results of some studies are contradictory, reflecting that the pro-survival or pro-death effect of mitophagy on cells or tissues is related to the difference in the type, timing, and extent of cells being stimulated and the surrounding microenvironment [86,87]. Therefore, how to reasonably regulate the level of mitophagy according to different diseases is a key direction of future research.

## Figures and Tables

**Table 1:** Mitophagy in IVDD.

Authors	Potential Targets	Cells	Signaling	Mitophagy	Function
Xie et al. [76]	CircERCC2	NPCs	miR-182-5p/SIRT1	Promoting	Alleviating IVDD
Wang et al. [77]	Moderate mechanical stress	NPCs	SIRT1-PINK1	Promoting	Alleviating IVDD
Xu et al. [19]	SIRT2	AF cells	SIRT2/PGC-1α	Inhibiting	Alleviating IVDD
Hu et al. [78]	/	NPCs	Nrf2/Sirt3	Promoting	Alleviating IVDD
Wang et al. [79]	honokiol	NPCs	AMPK/PGC-1α/SIRT3	Promoting	Alleviating IVDD
Lin et al. [80]	Urolithin A	NPCs	AMPK	Promoting	Alleviating IVDD
Wang et al. [81]	PINK1	NPCs	PINK1/Parkin	Promoting	Alleviating IVDD
Ma et al. [82]	SIRT1	NPCs	PINK1/Parkin	Promoting	Alleviating IVDD
Kang et al. [83]	MitoQ	NPCs	PINK1/Parkin	Promoting	Alleviating IVDD
Chen et al. [84]	Mfn2	NPCs	PINK1/Parkin	Inhibiting	Aggravating IVDD
Wang et al. [85]	MIF	NPCs	PINK1/Parkin	Promoting	Alleviating IVDD
Huang et al. [86]	Excessive mechanical stress	NPCs	PINK1/Parkin	Promoting	Aggravating IVDD
Lan et al. [87]	/	NPCs	Parkin	Promoting	Alleviating IVDD
Lin et al. [88]	LRRK2	NPCs	Parkin	Promoting	Alleviating IVDD
Chen et al. [89]	Melatonin	NPCs	Parkin	Promoting	Alleviating IVDD
Kang et al. [90]	/	Endplate chondrocyte	Parkin	Promoting	Alleviating IVDD
Zhang et al. [91]	Salidroside	NPC	Parkin	Promoting	Alleviating IVDD
Madhu et al. [28]	HIF-1	NPC	HIF-1α-BNIP3	Promoting	Alleviating IVDD
Xu et al. [92]	/	NPC	HIF-1α/NDUFA4L2	Promoting	Aggravating IVDD

## Data Availability

Not applicable.
